# Rethinking Feature Generalization in Vacant Space Detection

**DOI:** 10.3390/s23104776

**Published:** 2023-05-15

**Authors:** Hung-Nguyen Manh

**Affiliations:** Faculty of Electrical and Electronics Engineering, HCMC University of Technology and Education, Ho Chi Minh City 7000, Vietnam; hungnm@hcmute.edu.vn

**Keywords:** vacant space setection, information bottleneck, deep learning

## Abstract

Vacant space detection is critical in modern parking lots. However, deploying a detection model as a service is not an easy task. As the camera in a new parking is set up at different heights or viewing angles from the original parking lot where the training data are collected, the performance of the vacant space detector could be degraded. Therefore, in this paper, we proposed a method to learn generalized features so that the detector can work better in different environments. In detail, the features are suitable for a vacant detection task and robust to environmental change. We use a reparameterization process to model the variance from the environment. In addition, a variational information bottleneck is used to ensure the learned feature focus on only the appearance of a car in a specific parking space. Experimental results show that performances on a new parking lot increase significantly when only data from source parking are used in the training phase.

## 1. Introduction

Vacant space detection systems can help drivers quickly and easily find available parking spaces, reducing the time and frustration associated with circling a lot looking for a space. This can also help to reduce congestion in the parking lot, improve traffic flow, and reduce emissions from idling vehicles. Additionally, parking lot managers can use the data collected by these systems to optimize parking lot layouts and improve overall efficiency.

Recently, many sensors have been available for use in vacant space detection. For instance, ultrasonic sensors [1,2] use sound waves to detect the presence or absence of a vehicle in a parking space. They are typically installed above each parking space and can accurately detect the distance to the nearest object such as a vehicle. Magnetic sensors [3,4] use a magnetic field to detect the presence or absence of a vehicle in a parking space. They are typically installed beneath the surface of the parking space and can detect changes in the magnetic field caused by the presence of a vehicle. Infrared sensors [5] use infrared light to detect the presence or absence of a vehicle in a parking space. They are typically installed above each parking space and can detect changes in the infrared light caused by the presence of a vehicle. Video cameras [6,7,8,9,10,11] can be used to detect the presence or absence of a vehicle in a parking space. They are typically installed above each parking space and use image analysis software to detect changes in the video feed caused by the presence of a vehicle.

**Figure 1:**
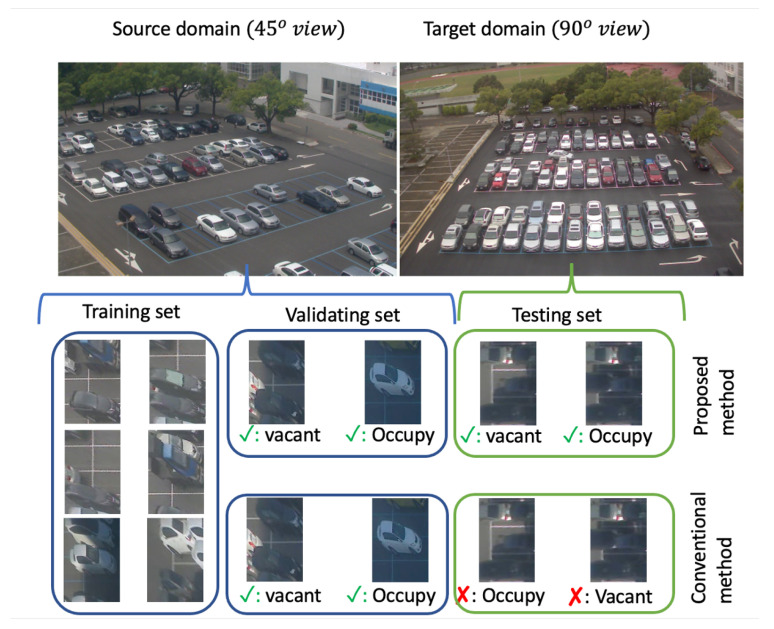
Performance of the vacant detector when training and testing on different parking lots. We set up two cameras to capture the same parking lot but at different angle views. These angle views represent the source and target domains. Samples are collected as a normalized three-parking slot Vu 2019 [12]. Training and evaluating datasets are collected on the source domain, and a testing dataset is collected on the target domain. The first row is the result of the proposed method; the second row is the conventional method’s result.

Among these possible sensors, many research works focus on vision-based systems to detect vacant spaces because a single camera can manage multi-parking slots. Several challenges should be addressed to have a workable vacant space detector, such as mutual occlusion patterns, shadow, layout, and size variances. From a literature review, supervised learning methods (Vu 2019 [12] and Huang 2017 [13]) require massive labeled data for a high accuracy of 99.74%. However, the domain gap could significantly reduce accuracy when the model is deployed in a new parking lot. As shown in Figure 1, conventional methods may make a wrong prediction in a new testing domain, while our method can learn generalized features that work robustly in a new testing domain.

Transfer learning [14] and unsupervised domain adaptation [15] are popular solutions to relieve the above-mentioned problem. Transfer learning [14] uses a pre-trained model to initialize the weight of the target network. As the pre-trained model is trained based on a vast dataset, its parameters could extract semantic information. Hence, the target model could converge faster with fewer training data. In the vacant space application, the pre-trained model could be trained based on data from a source parking lot; and the target network is a detector deployed in a new parking lot. Given a powerful pre-trained model, transfer learning requires enough newly labeled samples to fine-tune the target network to ensure success.

On the other hand, unsupervised domain adaptation [15] requires labeled data on a source domain and unlabeled data on a target domain. An adversarial loss [16] is used to train the sharing features between two domains, and labeled data in the source domain will be used to train a classifier based on the sharing features. To ensure the success of domain adaptation, the learned sharing features should be good enough for the specific task.

Both transfer learning and unsupervised domain adaptation need help from a pre-trained feature extractor trained on the source dataset. These features should be highly relevant to vacant states and invariant from environmental changes. Since different camera views create different occlusion patterns, learning in-variant features from an unseen domain (target domain) is challenging. To address the challenge, the paper tries to learn features that represent only objects (parking cars) but cannot present the background; even the background includes partial neighbor cars. We use mutual information [17] to measure the amount of information shared by (features and objects) as well as (features and backgrounds); the network is trained with an additional VIB loss.

**Figure 2:**
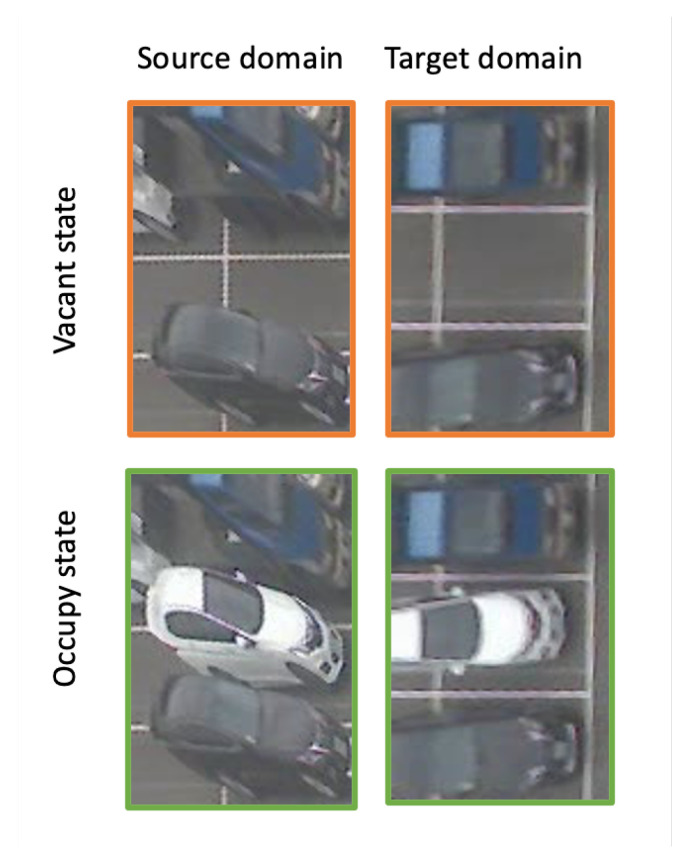
Vacant and occupied samples [12] on source and target domains. They are collected at the same slot and belong to a trajectory [18]. Each row represents samples at the same slot but from different domains. Each column represents samples in a trajectory where the background is similar, but the differences are from the object at the middle slot.

The idea behind the proposed method is quite similar to the concept in TCL2021 [18] regarding feature learning. TCL2021 [18] uses a trajectory with sequential frames to train the detector. The frames are selected before and after a car moves in/out of a slot; hence, they have pairwise vacant and occupied samples that share the same background but diffident vacant states. As shown in Figure 2, the pairwise samples in a trajectory had similar appearances; the only difference between the samples is at some pixels where the car is parked. These pairing data function as a contrastive loss where two samples in a trajectory are forced to move far from each other; hence, the model focuses on necessary pixels and eliminates all background pixels. While many promising results have been reported in TCL2021 [18], this method relies on a motion classifier to prepare training trajectories. Moreover, the training process needs a task-consistency reward to train the vacant-space detector in a reinforcement manner. This makes the training process more complex and requires powerful hardware to store all frames in a trajectory for every iteration update.

Unlike the complex training process in TCL2021 [18], our work learns crucial features using a simple process. Here, the model includes a feature extractor and a classifier. Given an input image *x*, the extractor extracts the feature $\mu \in R^{d}$, and the corresponding variance $\sigma \in R^{d}$. As the classifier must work well with variant features, we use a reparameterization trick to add some uncertainty to the training process. In detail, a latent feature *z* is sampled from the distribution $z∼N(\mu ,\;\sigma^{2})$; then, the vacant state *y* is predicted from sampled feature *z* by $y=cls(z;\theta_{cls})$.

A good feature *z* must represent occlusion patterns that predict well vacant states. Hence, the mutual information I(y;z) should be maximized. Additionally, the background information from input *x* should be eliminated in *z*. Hence, the mutual information I(x;z) should be small. Two constraints are optimized together in a variational information bottleneck (VIB) loss [19]. Since this loss could be integrated into any supervised learning framework, the training process becomes simpler than task consistency learning in TCL2021 [18].

In short, the contributions of this paper are the following:

- We propose a learning method to learn better features for vacant parking space detection. The trained model can work better on the unseen domains, while no data from the unseen domain are available in the training phase;
- A reparameterization trick is used to learn a classifier that is well adapted to environmental changes;
- A variational information bottleneck loss is used to learn features focused on occlusion patterns and eliminate the background.

## 2. Related Works

Vacant space detection for vehicle parking has been an interesting research topic for many years. Early research Lin 1997 [6], Raja 1999 [20], Raja 2000 [7], and Raja 2001 [21] relied on feature engineering processes to detect a car in a specific position. Given a selected location, Lin 1997 [6] introduces geometrical models with a spatial contour matching approach and a careful tuning process to fit well the specific scenario. Raja 1999 [20], and Raja 2001 [21] proposed a learning-based approach to model the unknown distribution of images that contain vehicles by utilizing the higher-order statistics (HOS) information as the feature. Raja 1999 [7] advanced to detect and track vehicles in videos by combining statistical knowledge about the visual appearance of vehicles with their motion information. However, most of the work focuses on a few slots but does not use only one camera to manage all slots in a wide parking area. This may limit particular applications.

With the development of CCTV systems, applying vehicle detection algorithms in the parking lot for vacant space detection has become possible in the recent decade. Therefore, much research has been conducted to pursue desirable performance and overcome challenging issues in practice (Paolo 2013 [22], Lixia 2012 [23], Wu 2007 [24], Huang 2010 [25], and Huang 2013 [26]). Here, images captured by CCTV cameras may include many slots. A detector must address both localization and classification tasks. Since a car should be parked in a specific 3D slot, prior 3D information could be used to address the localization task. In Huang 2010 [25], geometry and image information are fused to generate all vacant states of all slots. Additionally, neighbor slots help to correct the prediction at a query slot via Bayesian inference. Here, six 3D surfaces represent the 3D cube, and each surface is projected to a 2D image to find a corresponding region. Each region is processed independently, and a hierarchy Bayesian inference fuses predictions from these planes to a final vacant state. The Bayesian hierarchical structure can model the occlusion relationship among all neighboring slots to improve accuracy. However, the inference time of Huang 2010 [25] is too long, and the computing cost needs to be lowered to fit the real-world application. Later, in Huang 2013 [26], the Bayesian hierarchy is replaced by a multi-layer inference framework to learn the correction process. While Huang 2013 [26] presented multi-processing steps, Huang 2015 [27] models each processing step as a layer of a unique model. It helps the solution of Huang 2015 [27] to be simpler than the Bayesian hierarchy Huang 2013 [26]. Although considerable results are achieved, these methods require experts to tune hyper-parameters manually for each inference layer.

With the rapid development of deep learning techniques, many applications are solved using the deep model. The multi-layer in Huang 2015 [27] could be replaced by a unique deep learning model in Huang 2017 [13]. Each layer in Huang 2015 [27] is modeled by a specific block; later, the network is trained end-to-end. Following the experimental result in Huang 2017 [13], a detector may make a wrong prediction if a car is too big or parks not at the center of a slot. Therefore, in Huang 2017 [13], a spatial transform network is used to select a suitable region of the input image for classification. To ensure the input image can cover all necessary information so that a spatial transform could select a suitable ROI, the authors of Huang 2017 [13] used a normalized three-parking image to represent input and gain higher accuracy in real-time. Next, Vu 2019 [12] provides a contrastive loss to learn better feature representation. Using a deep learning framework, the methods in Huang 2017 [13] and Vu 2019 [12] perform better than traditional approaches without fancy feature engineering processes or heavy hyper-parameter adjustment. To deploy the vacant detector in a new parking lot, Zeng 2022 [28] further generalized the deep learning-based approaches to significantly different lighting scenarios with the adversarial domain adaptation technique. Both Vu 2019 [12] and Zeng 2020 [28] reused the normalized three-parking-space image setting in Huang 2017 [13] and obtained high-accuracy results.

Although promising results were reported in Vu 2019 [12], and Zeng 2022 [28], the success of these deep learning approaches comes from a vast dataset. Unfortunately, due to the domain gap issue, using these approaches requires labeling once for each parking lot, which causes enormous manual effort. To solve this issue, TCL2021 [18] proposes using an optical-flow-based motion classifier as the guidance to train the vacant space detector. Specifically, TCL2021 [18] found that RGB-image-based models are affected seriously by camera poses or various lighting conditions. By contrast, the motion classifier, which takes a sequence of optical flow to predict the motion state of a trajectory, is robust to such factors. Therefore, the authors train a vacant space detector using consistency with a flow-based motion classifier. The motion classifier could be trained in a parking lot; later, it helps to train vacant detectors in another parking lot. Using consistency between the two models, the vacant space detector on a new parking lot could be trained without human labor. However, the motion classifier may make a wrong prediction with high confidence; this phenomenon seriously affects the trained detector. Moreover, estimating a consistency reward is complicated, and this method requires high-capacity hardware to store all frames in a trajectory.

## 3. Proposed Method

### 3.1. Problem Statement

Given a parking lot monitored by a surveillance camera mounted at a high position, we would like to train a vacant space detector to detect a vacant state at any slot in the parking space. However, unlike the conventional methods (Huang 2017 [13], and Vu 2019 [12]), where training and testing images are from the same parking lot, our work uses training images and testing images from different domains. Figure 1 shows the images captured from two domains (different angle views). The domain gap by camera poses results in different appearances in the same parking lot.

Conventionally, a detector must address localization and classification tasks. In a vacant space detector, the localization task could be well addressed by the 3D position of a parking slot. Huang 2017 [13] used a 3D cube to model each slot, and the projection of the cube to a 2D image serves as a localization result. Hence, we can focus on the classification task provided by the local patch. Following a careful design, Vu 2019 [12] introduced a three-space normalization as an input of the classifier. Here, three neighbor slots are concatenated and projected on a 2D image. The projection is normalized as an input of a classifier. Some examples of the normalization samples are shown in Figure 2.

Denote as $D^{S}={\left\{x_{i}^{s},y_{i}^{s}\right\}}_{i=1\ldots N}$ a dataset collected on the source domain in Figure 1. We separate it into training and validating datasets $\left(D_{Train}^{S},D_{Val}^{S}\right)$. A detector is trained and validated using these datasets; then, the detector is tested on a testing dataset $D_{Test}^{T}$. Here, $D_{Test}^{T}$ is collected on the target domain using the same 3-space-normalization method. Note that $D_{Train}^{S}$ and $D_{Test}^{T}$ are collected on different days, and no image in the target domain is used during the training process. If the trained model predicts well in the target dataset, the method could learn generalized features invariant to the camera angle. Some examples of $D_{Train}^{S}$ and $D_{Test}^{T}$ are in Figure 2.

### 3.2. System Overview

**Figure 3:**
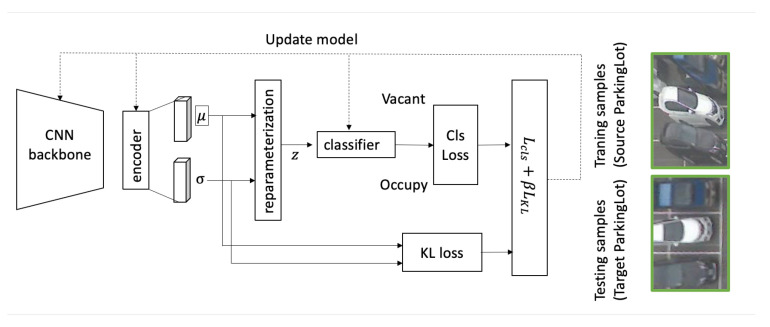
System overview.

Figure 3 presents the overview of the proposed method. First, a pre-trained model extracts flattened feature $f=CNN\left(x\right)\in R^{D}$. The features are fed forward to an encoder to generate $\mu \in R^{d}$ and $\sigma \in R^{d}$ vectors. Two vectors are used to estimate the Kullback–Leibler divergence loss [29]; additionally, a reparameterization process samples a new latent *z*. A classifier takes the latent *z* and predicts the vacant state y∈{vacant,occupy}.

The paper uses VGG16 [30] as a backbone (feature extractor and neck) to provide a 512×7×7 feature map. However, the extractor could be any pre-trained backbone. In the encoder module, linear layers encode the flattened feature to a μ,σ. Table 1 summarizes the network architecture.

### 3.3. Variational Information Bottleneck (VIB)

#### 3.3.1. Overview of VIB Loss for Vacant Space Detector

Conventionally, a deep neuron network uses an extractor to map input image *x* to a feature space *z*; the feature is fed to a classifier to predict an output state *y*. A generalized feature should be invariant to environmental factors so the classifier can predict well in the testing phases. In a vacant space detector, learning generalized features is a very challenging task. As shown in Figure 2, the vacant and occupied samples are similar because they share the same background. Both of them include cars in the images; the only difference is that occlusion patterns may occur while a car is parked in the middle slot. The conventional method may learn many features (e.g., car patterns) and use the pattern to predict the vacant state. These features can work well to predict the vacant state in the same domain but will be affected if the domain changes (e.g., an angle view). A good feature for vacant space detection is a very compressed feature that focuses only on the occlusion pattern in the mid-slot.

The information bottleneck principle [31] introduces a view of supervised learning to learn these good features. The principle says that the optimal model transmits as much information as possible from its input to its output through a compressed representation called the information bottleneck. In vacant space detectors, the information bottleneck could be explained by the following two constraints:

- The latent *z* must help to predict well the output *y* (vacant state);
- The latent *z* must be compressed to the input *x* (3-space normalization images). This means that when we know *z*, we cannot infer *x* very well.

(1) $$\begin{array}{cc} I(X;Y) & =I(Y;X)\\ \; & =H(X,Y)-H(X|Y)-H(Y|X)\\ \; & =H(X,Y)-H(X|Y)-H(Y|X)\\ \; & =KL{\left(p(x,y)||p(x)p(y)\right)}_{x\in X,y\in Y}\\ \; & =E_{\left(x,y)∼p(x,y\right)}\left[\log\frac{p(x,y)}{p(x)p(y)}\right]\\ \; & =\int p\left(x,y\right)\log\frac{p(x,y)}{p(x)p(y)}dxdy. \end{array}$$

From information theory, the mutual information [17] can measure the information of a variable via another variable as in Equation (1). Hence, the above constraints could be explained by maximizing the I(y;z) and minimizing I(x;z). Denote as $I_{c}$ the information constraint that $I\left(x;z\right)<I_{c}$; the Lagrangian of the above-constrained optimization problem is a maximization solution of Equation (2). Here, β is a Lagrange multiplier.

(2) $$\begin{array}{cc} L_{IB} & =I\left(y;z\right)-\beta \left(I\left(x;z\right)-I_{c}\right)\\ \; & \approx I(y;z)-\beta I(x;z). \end{array}$$

#### 3.3.2. Classification Loss in VIB

By applying the definition in Equation (1), the first term I(y;z) could be rewritten as Equation (3). Here, p(y∣z) is defined by an intractable function as Equation (4).

(3) $$\begin{array}{cc} I(y;z) & =\int p\left(y,z\right)\log\frac{p(y,z)}{p(y)p(z)}dydz=\int p\left(y,z\right)\log\frac{p(y|z)}{p(y)}dydz\\ \; & =\int p(y,z)\log p(y|z)dydz-\int p(y)\log p(y)dy. \end{array}$$

(4) $$p\left(y|z\right)=\frac{p(y,z)}{p(z)}=\int \frac{p(x,y,z)}{p(z)}dx=\int \frac{p(z|x)p(y|x)p(x)}{p(z)}dx.$$

Since Equation (4) is intractable, the authors of [19] used a lower bound to approximate p(y∣z). Denote as q(y∣z) a variational approximation of p(y∣z); the lower bound is founded by Kullback–Leibler divergence as in Equation (5).

(5) $$KL\left(p(y|z)||q(y|z)\right)\geq 0⟹\int p\left(y|z\right)\log p\left(y|z\right)dy\geq \int p\left(y|z\right)\log q\left(y|z\right)dy.$$

Using the lower bound in Equation (5), the I(y;z) in Equation (3) is rewritten as Equation (6).

(6) $$\begin{array}{cc} I(y;z) & \geq \int p(y,z)\log q(y|z)dydz-\int p(y)\log p(y)dy\\ \; & \approx \int p(z|x)p(y|x)p(x)\log q(y|z)dxdydz\\ \; & \approx \int p(z|x)p(y,x)\log q(y|z)dxdydz. \end{array}$$

where the entropy of the labels H(y)=−∫p(y)logp(y)dy is independent and could be ignored.

#### 3.3.3. Feature Selection Loss in VIB

The second term I(x;z) is rewritten as Equation (7).

(7) $$I\left(x;z\right)=\int p\left(x,z\right)\log\frac{p(x,z)}{p(x)p(z)}dxdz=\int p\left(x,z\right)\log\frac{p(z|x)}{p(z)}dxdz.$$

Let q(z) be a variational approximation to the marginal p(z). Using the KL divergence, the upper bound of I(x;z) is introduced as Equation (8).

(8) $$KL\left(p(z)||q(z)\right)\geq 0⟹\int p\left(z\right)\log p\left(z\right)dz\geq \int p\left(z\right)\log q\left(z\right)dz.$$

Applying the upper bound to Equation (7), the I(x;z) is rewritten as Equation (9).

(9) $$\begin{array}{cc} I(x;z) & =\int p(x,z)\log p(z|x)dxdz-\int p(z)\log p(z)dz\\ \; & \leq \int p(x,z)\log p(z|x)dxdz-\int p(z)\log q(z)dz\\ \; & =\int p\left(x\right)p\left(z|x\right)\log\frac{p(z|x)}{q(z)}dxdz\\ \; & =\int p\left(x,y\right)p\left(z|x\right)\log\frac{p(z|x)}{q(z)}dxdzdy. \end{array}$$

#### 3.3.4. Approximation Loss Function

By applying the lower bound of the I(y;z) and the upper bound of I(x;z), the Lagrangian function in Equation (2) is approximated as

(10) $$\begin{array}{cc} L_{IB} & =I(y;z)-\beta I(x;z)\\ \; & \geq \int p\left(z|x\right)p\left(y,x\right)\log q\left(y|z\right)dxdydz-\beta \int p\left(z|x\right)p\left(x,y\right)KL\left(p(z|x)||q(z)\right)dxdydz\\ \; & =E_{\left(x,y)∼p(x,y),z∼p(z|x\right)}\left[\log q\left(y|z\right)-\beta KL\left(p(z|x)||q(z)\right)\right]\\ \; & =J_{IB}. \end{array}$$

In a practical application where the dataset ${\left(x_{i},y_{i}\right)}_{i=1..N}$ is available, the $J_{IB}$ is estimated as Equation (11).

(11) $$J_{IB}=\frac{1}{n}\sum_{i=1}^{n}\left[\int p\left(z|x_{i}\right)\log q\left(y_{i}|z\right)dz-\beta KL\left(p\left(z|x_{i}\right)\left|\right|q\left(z\right)\right)\right].$$

To model the loss $J_{IB}$ as a deep learning framework, ref. [19] use an encoder Φ to predict $\mu_{\Phi }\left(x\right),\sum_{\Phi }\left(x\right)$. Then, $p_{\Phi }\left(z|x\right)$ is modeled as a multivariate Gaussian $p_{\Phi }\left(z|x\right)=\;$$N\left(z;\mu_{\Phi }\left(x\right),\sum_{\Phi }\left(x\right)\right)$. The latent *z* could be sampled from a reparameterization trick g(ϵ,x) where ϵ∼p(ϵ)=N(0,I). Thus, the final objective is a minimized solution of the loss function in Equation (12). Here, *n* is the number of training samples.

(12) $$J_{IB}=\frac{1}{n}\sum_{i=1}^{n}\left[\beta KL\left(p_{\Phi }\left(z|x_{i}\right)\left|\right|q\left(z\right)\right)-E_{\epsilon ∼p(\epsilon )}\left[\log q\left(y_{i}|g\left(\epsilon ,x_{i}\right)\right)\right]\right].$$

Here, the term q(y∣g(ϵ,x)) serves as a classifier and it is learned by a binary cross entropy loss as Equation (13), whereas q(z) is the approximated latent marginal often fixed to a standard normal distribution as Equation (14)

(13) $$-\log q\left(y|g\left(\epsilon ,x\right)\right)=-\left[y\log\hat{y}+\left(1-y\right)\log\left(1-\hat{y}\right)\right].$$

(14) $$q=N\left(z;0,I_{k}\right).$$

## 4. Results

### 4.1. Datasets and Implementation Detail

**Figure 4:**
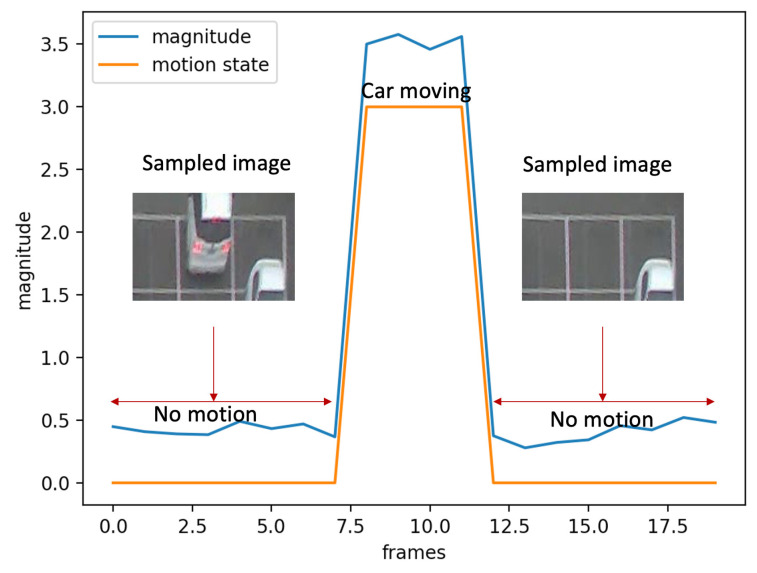
The process of collecting training data in TCL2021 [18]. The X-axis is the frame index; the Y-axis is the optical flow magnitude.

Two training datasets are collected on the source domain in Figure 1. The first training dataset $D_{Train_{1}}^{S}$ is a standard training dataset collected over 30 days and labeled for supervised learning. We collect one image every 30 min and only in the daytime—from 6 a.m. to 6 p.m. The dataset includes 14,667 vacant slots and 20,021 occupied slots. The second training dataset $D_{Train_{2}}^{S}$ includes samples collected from trajectories as in TCL2021 [18]. In detail, TCL2021 [18] uses the magnitude of optical flow to detect a time slot when a car is moving. Given the optical flow in the time slot, a motion classifier is used to estimate the motion state of this car. On both sides (before and after) of the time slot, we have no-motion segments in which no car moves, as in Figure 4. We sample one sample for each no-motion segment. Then, two samples are added to the $D_{Train_{2}}^{S}$ dataset. As a result of the sampling method, the pair samples have similar backgrounds but only differences in the middle slot, as shown in Figure 2 and Figure 4. This dataset includes 2000 samples from 1000 trajectories. Hence, $D_{Train_{1}}^{S}$ is a vast and diverse dataset that may make it easy to train a vacant space detector; in contrast, $D_{Train_{2}}^{S}$ is small but has many similar images in the dataset. This property allows $D_{Train_{2}}^{S}$ to work as a contrastive loss where pairwise samples are trained together.

In the testing phase, two datasets are prepared. The first dataset $D_{Val}^{S}$ includes 11,944 vacant samples and 7832 occupied slots collected from the other 15 days on the source parking lot from 6 am to 6 pm. The second dataset $D_{Test}^{T}$ is of the same scale as the $D_{Val}^{S}$ dataset but it is collected from the target domain. We use the two testing datasets to evaluate the effects of the domain gap and the generalization of models trained by the proposed method.

We follow the suggestion in [32] to implement the VIB method. However, instead of using a sum operator to estimate the loss over training patches, we use a mean operator. Given an input *x* and the corresponding latent $z\in R^{d}$, the sum operator estimates KL loss *d* times, but the classification loss is only estimated once for each sample. In this situation, gradients from the KL loss may destroy the original backbone. Using the mean operator, the losses are averaged on both the feature and patch size dimensions. Hence, the model can converge smoothly.

In addition, the training hyper-parameters are listed below:

- Optimizer: SGD with learning rate = 0.01.
- Scheduler: step_size = 30 and gamma = 0.1.
- Data normalization: mean = [0.485, 0.456, 0.406]; std = [0.229, 0.224, 0.225].

### 4.2. Ablation Study

In this section, we discuss the contribution of the KL loss on feature learning. The quality of features depends on the quality of the training dataset. Therefore, we randomly select a subset of $D_{Train_{1}}^{S}$ to train the detector. Denote as $S_{1}$, $S_{2}$, $S_{3}$, $S_{4}$, and $S_{5}$ subsets from $D_{Train_{1}}^{S}$. The number of samples in $S_{1}$, $S_{2}$, $S_{3}$, $S_{4}$, and $S_{5}$ is 2000, 5000, 10,000, 20,000, and 34,688, respectively. As the $S_{1}$ dataset has 2000 samples, it is equivalent to $D_{Train_{2}}^{S}$ in scale. However, differences are in the content of $D_{Train_{2}}^{S}$ where pairwise vacant/occupy images share the same background.

We first use subsets of $D_{Train_{1}}^{S}$ to evaluate the generalization of a model. In this scenario, the training dataset is diverse. The accuracy values for $D_{Val}^{S}$ and $D_{Test}^{T}$ are in Table 2 and the F1 score for $D_{Val}^{S}$ and $D_{Test}^{T}$ are in Table 3. During the training process, we evaluate the model using $D_{Val}^{S}$ for every epoch. The accuracy for $D_{Val}^{S}$ is used to select the best model in the training phase.

Regularization methods (e.g., batch normalization, dropout) are possible solutions to avoid overfitting on a dataset and learn generalization features. Hence, we use batch normalization and dropout on the conventional supervised learning method to evaluate regularization methods on the application. Our experiment is based on the VGG model to detect vacant space. The VGG model includes a feature extractor, a neck, and a classifier. Hence, we add a batch normalization after the feature extractor and denote this setting as “No VIB (BN)” in Table 2 and Table 3. In the classifier module (VGG network), the dropout layer had been applied with *p* = 0.5 by default. Hence, the default setting is denoted as “No VIB (*p* = 0.5)”. To attain better generalization features, we test the performance when *p* = 0.7 and *p* = 0.9. The notations of these settings are “No VIB (*p* = 0.7)” and “No VIB (*p* = 0.9)”, correspondingly.

Following the result in Table 2 and Table 3, several conclusions could be drawn:

- The domain gap is a critical challenge in deploying a new parking lot. In Table 2, using the $S_{2}$ dataset (5000 samples), the conventional method (No VIB (p=0.5)) can achieve 99.46% accuracy on $D_{Val}^{S}$ but it can only achieve 89.68% accuracy on $D_{Test}^{T}$;
- To learn generalized features, a vast dataset is needed. In the scenario “No VIB (p=0.5)” in Table 2, using the $S_{5}$ dataset (30,540 samples) for training does not help to increase accuracy when evaluating on the same domain ($D_{val}^{S}$). However, the performance increases significantly when evaluating is based on the target domain ($D_{test}^{T}$). In detail, when the $S_{2}$ dataset is used for training, the accuracy on $D_{test}^{T}$ is only 89.68%; however, when the $S_{5}$ dataset is used for training, the corresponding accuracy is 92.02%. A similar observation can be found in Table 3;
- The proposed method helps to increase the performance on the source domain if the training dataset is sparse. When the training set is $S_{1}$ dataset (2000 samples), the accuracy and F1-score are 97.95% and 97.54%, correspondingly, on $D_{Val}^{S}$. When the KL-loss (β=0.1) is introduced, the performance improves to 98.44% for accuracy and 98.14% for the F1-Score on the source domain;
- When the number of training samples increases, the proposed method helps to learn better features that work well in the target domain. The conventional model (No VIB (p=0.5)) can reach up to 88.95% on the $D_{Test}^{T}$ dataset. However, there are no apparent differences between the accuracies given by the $S_{3}$ (only 10,000 samples) and $S_{5}$ datasets. On the target domain, the accuracy given by $S_{3}$ is 88.84% and the accuracy given by $S_{5}$ is 88.95%. In contrast, given the $S_{5}$ datasets, the proposed method can achieve 97.91% accuracy and 97.31% F1 score (β=0.1). Moreover, the differences in accuracy are apparent if more training samples are available. We can see that the performance inmproves if the number of training samples is increased. In further detail, when $S_{1},S_{2},S_{3},S_{4},S_{5}$ are applied for training, the accuracies are 92.93%, 94.73%, 95.28%, 96.51%, 97.91%, correspondingly;
- The hyper-parameter β should not be too large. When β=0.2, the performance is reduced on $D_{Test}^{T}$. This is reasonable because the KL-loss forces a feature to be closer to zero. This means that more spatial regions are not used to predict the vacant state. Following our experiment, β=0.1 is an optimal selection in most datasets;
- Batch normalization may not help in the vacant space detection application. Even if the performance on the source domain is high, the model cannot work better on the target domain. The maximum accuracy on the target domain is only 83.53% if BN is applied;
- The increment of the dropout parameter may help with small training sets. If $S_{1}$ or $S_{2}$ serve as training sets, the performance improves on both source and target domains. Especially when the $S_{2}$ dataset is used for training and the dropout ratio is 0.9, the conventional method performs better than our proposed method. However, when the dataset is larger, the performance cannot improve. The accuracies on the $S_{2}$, $S_{3}$, $S_{4}$, and $S_{5}$ datasets are quite similar if p = 0.5 or p = 0.7. In contrast, our VIB-based method can also learn and accept new features from larger training datasets.

We also provide experiments to evaluate the proposed method if the training dataset includes pairwise samples. Here, $D_{Train_{2}}^{S}$ serves as a training set and the testing sets are $D_{Val}^{S}$ and $D_{Test}^{T}$ as in previous experiments. Note that pairwise samples in $D_{Train_{2}}^{S}$ serve as a contrastive loss during the training phase. The result in Table 4 shows that a pairwise dataset may help to learn better-generalized features. Compared with the result given by the $S_{1}$ dataset, the result given by the $D_{Train_{2}}^{S}$ dataset is slightly better. The accuracy scores are 98.04% and 97.95% if the training datasets are $D_{Train_{2}}^{S}$ and $S_{1}$, correspondingly. Here, we compare the two datasets because they are on the same scale. The pairwise dataset [18] $D_{Train_{2}}^{S}$ could help to slightly increase performance. This phenomenon could also be observed when $D_{Test}^{T}$ is the testing set. Hence, the pairwise property in $D_{Train_{2}}^{S}$ can force the model to learn generalized features.

When applying VIB, the performance on the source domain ($D_{Val}^{S}$) is not improved. The accuracy is 98.04% without VIB and it is 98.97% with VIB. This means $D_{Train_{2}}^{S}$ does not have enough data to learn a better detector. This conclusion is consistent with the result in Table 2 when $S_{1}$ is the training set. However, the performance of the target domain is improved significantly. Without VIB, the accuracy on $D_{Test}^{T}$ is 85.88% but it could increase to 94.82% when β=0.1. In this case, the increment is 8.94%. If the $S_{1}$ dataset is used as a training set, the increment is 8.75%. This means that VIB loss can help in both types of datasets.

### 4.3. Comparison with State-of-the-Art Methods

#### 4.3.1. Comparison with Supervised Learning Methods

In addition, we compare our method with other conventional vacant space detectors, including [12,13,18,22,23,24,33]. The parking lot in our paper is similar to the parking lot in Vu 2019 [12] and TCL2021 [18]. However, Vu 2019 [12] prepared a vast dataset that includes 587,667 samples and TCL2021 [18] used a motion classifier to select pairwise samples. The two papers and our paper use a normalized three-parking slot as the input of detectors. The authors of Vu 2019 [12] also used their dataset to evaluate Paulo2013 [22], Lixia 2012 [23], Wu 2007 [24], Huang2017 [13], and Faster-RCNN [33] methods in a comprehensive comparison. From a dataset viewpoint, our dataset is quite similar to the dataset in Vu 2019 [12] but at a smaller scale. From the training process viewpoint, Vu2019 [12] does not use any pre-trained model, but the proposed method uses the pre-trained VGG model. We also prepared a simple version of the proposed method that does not use VGG as a pre-trained model.

The comparison results in Table 5 show that our method can outperform these vision-based methods as [22,23,24]. Moreover, we also compared with deep-learning-based methods [12,13,33]. Faster-RCNN [33] works for vehicle detection, but it cannot detect well occluded parked cars with small image sizes. In addition, Faster-RCNN needs to address both the localization and classification tasks, while Huang 2017 [13] and Vu 2019 [12] use 3D information to solve the localization task. Hence, their performances are better. Huang 2017 [13] and Vu 2019 [12] can provide higher accuracy for vacant space detection, but they require a vast training dataset with supervised labels. In comparison, our method also achieves an equivalent accuracy with only 5000 samples. However, Huang 2017 [13] and Vu 2019 [12] do not use any pre-trained model, but our method uses a VGG pre-trained model. For a better comparison, we also train our method from scratch with only 5000 training samples. The result shows that without the help from the VGG pre-trained model, the accuracy is reduced by 1%. This degradation is relatively small and could be compensated for when the training dataset is larger. TCL [18] uses 1000 motion trajectories to achieve a good performance, but their training process is complex because of the task consistency reward. Additionally, it requires more RAM in GPU to store all frames in a trajectory. Compared with TCL [18], our proposed method is easier to train.

#### 4.3.2. Comparison with an Upper Bound

In our work, we train and evaluate the vacant space detector on the source domain; additionally, we test it on the target domain. During the training process, no information from the target domain is used. To evaluate our learning method’s performance, we compare the proposed method with unsupervised domain adaptation [28]. In unsupervised domain adaptation [28], the target model uses a source dataset and a target dataset during training phases. However, the source dataset includes label and image information, whereas the target dataset only has image information. Therefore, the unsupervised domain adaptation [28] could be treated as an upper bound of our proposed method.

In this experiment, two cameras at two angle views are set up to collect two datasets. One dataset is the source dataset and another is the target dataset. Each dataset is split into a training dataset and a validation dataset in its domain. The results in Table 6 show that when ‘$45^{{}^{\circ}}$ view’ is used as a source dataset, the performance on the target dataset (‘$90^{\circ }$ view’) reaches the upper bound. However, if ‘$90^{\circ }$ view’ serves as the source dataset, the performance on ‘$45^{{}^{\circ}}$ view’ is far from the upper bound. This means that generalized features rely greatly on the source domain.

### 4.4. Feature Analysis

**Figure 5:**
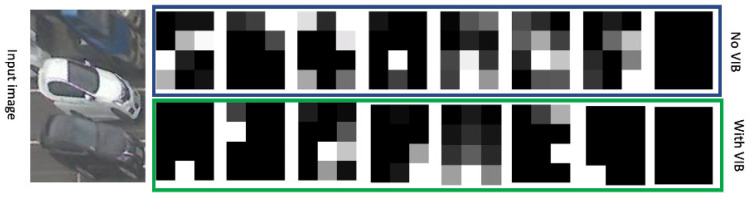
Feature maps extracted by the detector with and without VIB loss.

In this section, we analyze the feature maps extracted by the proposed method (with VIB) and the conventional method (without VIB). Our model is based on the VGG model that includes a feature extractor, a neck, and a classifier. Given an image, we use the feature extractor to extract feature maps. There are 512 feature maps given one input image. Figure 5 shows one example image and several corresponding feature maps in both cases (with and without VIB). The result shows that the VIB-based method extracts sparse feature maps. This means that the features learned by VIB are precise and cannot be found everywhere on the example image even though cars are at every neighbor slot. Only some spatial locations have a response on the feature map yielded by the VIB-based method. This phenomenon is reasonable because the KL loss force feature maps close to zeros. Similar to the dropout technique, a sparse feature map may avoid overfitting in a dataset and the model becomes generalized. This observation also proves the benefit of the proposed method where the model can serve as a feature selector which:

- Adaptively selects features suitable for the target task;
- Skips or removes redundancy features related to input images.

In addition, the proposed method can not only provide sparse feature maps but also extract more empty feature maps where all values are zeros. Given one feature map, we extract the feature map’s minimum, maximum, and mean values. This process is applied to 512 channels. The statistics of these variables are in Table 7. Each column represents one variable (maximum, minimum, and mean value of a feature map), and each row represents one statistics indicator.

By comparing the statistics of the maximum variable between the two methods (with and without VIB), we may see that the number of empty feature maps in the VIB-based method is larger. With VIB, more than 50% of feature maps are empty maps; without VIB, the corresponding value is smaller than 50%. In addition, there is not a huge distance between VIB-based features. With VIB, the maximum values of minimum variables and maximum variables are 7.3204 and 12.6495. Without VIB, the maximum values of minimum variables and maximum variables are 1.2250 and 30.8568. Considering the maximum value, the gap between maximum and minimum variables is 5.3291 and 29.6318 with VIB and without VIB. This means that VIB can serve as a normalization process that reduces the feature variance in feature space.

Following the above discussions, VIB has feature normalization and sparse representation properties. Normalization and sparse representation are well-known solutions for a better generalization model. Hence, the proposed method can learn generalization features. In addition, during the training process, some uncertainty was added to the feature by the reparameterization step. This uncertainty may model some domain shift factors (orientation, camera field of view, camera height) but in feature space. Therefore, the model has the ability to adapt to environmental change.

## 5. Conclusions

In this paper, we apply VIB [19] to learn generalized features for vacant space detectors. The training/validating datasets are from the source domain, while the testing dataset is from the target domain. Our method is significantly better than conventional methods if the detector is tested on the target domain. Additionally, our method helps improve performance on the source domain if the training dataset is sparse.

## Figures and Tables

**Table 1:** Network detail. Here, $F_{B}$ = 25,088, $F_{C}=2048,d=1024$.

Block	Layer	Parameters
${\mathrm{Encoder}}_{1}$	Flatten	Optional
${\mathrm{Encoder}}_{2}$	nn.Linear	In = $F_{B}$, Out = $F_{C}$
${\mathrm{Encoder}}_{3}$	nn.ReLu	Inplace = True
${\mathrm{Encoder}}_{4}$	nn.Linear	In = $F_{C}$, Out = 2d
Re-parametrize	z=μ+ϵ∗σ	$\epsilon ∼N(0,\sigma^{2})$
Classifier	nn.Linear	In = *d*, Out = 2

**Table 2:** The accuracy on source/target domain under different datasets and various β values. (The unit is %). The bold represents the best performance in each column.

Scenario	$S_{1}$	$S_{2}$	$S_{3}$	$S_{4}$	$S_{5}$
No VIB (BN)	93.43/76.13	98.82/75.16	99.47/83.45	99.68/83.53	99.78/82.47
No VIB (p=0.5)	97.95/84.18	99.46/89.68	99.62/91.93	99.62/92.02	99.63/92.02
No VIB (p=0.7)	98.18/87.63	99.46/92.52	99.70/94.49	99.79/94.76	99.84/95.88
No VIB (p=0.9)	96.75/88.89	99.41/**95.07**	99.63/95.01	99.81/95.46	99.63/95.76
β=0.05	98.01/90.88	99.19/92.86	99.36/93.77	99.60/94.26	99.80/96.06
β=0.07	98.25/90.12	99.22/94.71	99.50/**95.28**	99.69/95.21	99.77/97.02
β=0.1	98.21/**92.93**	99.29/94.73	99.51/94.75	99.79/**96.51**	99.81/**97.91**
β=0.2	98.44/89.26	99.41/92.09	99.51/92.69	99.78/93.48	99.81/94.53

**Table 3:** The F1 score on source/target domain under different datasets and various β values. (The unit is %). The bold represents the best performance in each column.

Scenario	$S_{1}$	$S_{2}$	$S_{3}$	$S_{4}$	$S_{5}$
No VIB (BN)	92.30/57.90	98.58/54.53	99.37/74.23	99.62/74.14	99.73/72.27
No VIB (p=0.5)	97.54/75.45	99.35/85.22	99.54/88.84	99.54/88.97	99.55/88.95
No VIB (p=0.7)	97.81/82.20	9935/89.73	99.64/92.78	99.75/94.73	99.80/95.58
No VIB (p=0.9)	96.10/84.37	99.28/**93.71**	99.56/94.06	99.77/94.05	9984/94.45
β=0.05	97.63/89.08	99.01/91.54	99.23/91.80	99.52/93.09	99.76/95.05
β=0.07	97.92/88.34	99.06/93.55	99.40/**94.22**	99.63/94.08	99.72/96.21
β=0.1	97.87/**91.56**	99.15/93.58	99.41/93.62	99.77/**95.62**	99.77/**97.39**
β=0.2	98.14/86.55	99.29/90.49	99.40/91.27	99.73/91.15	99.73/92.70

**Table 4:** Accuracy and F1-Score on source and target domains. The training dataset is $D_{train_{2}}^{S}$. (Unit is %). The bold represents the best performance in each column.

Domain	Metric	β=0.05	β=0.07	β=0.1	β=0.2	No VIB ($D_{{\mathrm{Train}}_{2}}^{S}$)	No VIB ($S_{1}$)
Source	ACC	98.68	**98.79**	98.77	98.65	98.04	97.95
$\left(D_{Val}^{S}\right)$	F1	98.04	**98.54**	98.52	98.36	97.61	97.54
Target	ACC	92.42	93.21	**94.82**	93.78	85.88	84.18
$\left(D_{Test}^{T}\right)$	F1	90.34	91.07	**93.28**	92.18	79.85	75.45

**Table 5:** Comparison to state-of-the-art vacant detectors on the same parking lot. Here, a sample refers to a normalized 3-parking-slot image and a trajectory refers to a video sequence when a car moves in/out of a slot. The bold represents the best performance in each column.

Method	# of Training Samples	Accuracy (%)
Paulo 2013 [22]	587,667 samples	81.98
Lixia 2012 [23]	587,667 samples	83.46
Wu 2007 [24]	587,667 samples	92.26
Huang 2017 [13]	587,667 samples	98.44
Vu 2019 [12]	587,667 samples	99.68
Faster-RCNN [33]	587,667 samples	72.68
TCL 2021 [18]	1000 trajectories	99.54
Proposed method (β=0.1) with VGG	5000 samples	**99.81**
Proposed method (β=0.1) no VGG	5000 samples	98.82

**Table 6:** Accuracy comparison with unsupervised domain adaption. (The unit is %.) (‘$90^{{}^{\circ}}$ view’ ⇒ ‘$45^{{}^{\circ}}$ view’) means that ‘$90^{{}^{\circ}}$ view’ is the source domain and ‘$45^{{}^{\circ}}$ view’ is target domain. Each experiment is repeated five times to obtain the min, mean, and max accuracies.

	‘$90^{{}^{\circ}}$ view’ ⇒ ‘$45^{{}^{\circ}}$ view’	‘$45^{{}^{\circ}}$ view’ ⇒ ‘$90^{{}^{\circ}}$ view’
Proposed method (min)	94.58	97.20
Proposed method (mean)	95.03	97.82
Proposed method (max)	95.13	98.25
Unsupervised Domain Adaptation [28]	98.2	98.85

**Table 7:** Statistics of maximum, minimum, and mean value on feature maps.

	With VIB			Without VIB		
	Maximum	Minimum	Mean	Maximum	Minimum	Mean
count	512	512	512	512	512	512
mean	0.4024	0.0750	0.1967	2.5487	0.0024	0.6033
std	1.1616	0.4952	0.8168	3.9279	0.0541	1.2015
min	0	0	0	0	0	0
25%	0	0	0	0	0	0
50%	0	0	0	0.8348	0	0.0748
75%	0.1759	0	0	3.5644	0	0.6591
max	12.6495	7.3204	9.8290	30.8568	1.2250	10.0369
